# Lung maneuvers of large amplitudes for probing physiological alterations in mouse models of asthma

**DOI:** 10.1002/ame2.70174

**Published:** 2026-03-10

**Authors:** Magali Boucher, Cyndi Henry, Marie‐Josée Beaulieu, Andrés Rojas‐Ruiz, Ynuk Bossé

**Affiliations:** ^1^ Institut Universitaire de Cardiologie et de Pneumologie de Québec (IUCPQ)—Université Laval Québec Québec Canada

**Keywords:** animal model, asthma, lung volumes, oscillometry, respiratory mechanics

## Abstract

**Background:**

Mouse models are commonly used to study asthma. Oscillometry, a technique using small‐amplitude maneuvers, is often used to probe physiological lung alterations in mice. However, the changes in oscillometric readouts in mouse models of asthma are typically subtle, implying the need for using large sample sizes. Herein, lung maneuvers of different amplitudes were used to compare their sensitivity in detecting physiological alterations in mouse models of asthma.

**Methods:**

Female BALB/c mice were exposed to saline or house dust mite (HDM) intranasally to induce experimental asthma. They were exposed either thrice per week for 5 weeks (long protocol) or for 10 consecutive days (short protocol). The presence of physiological alterations was tested using lung maneuvers of small amplitudes (oscillometry), intermediate amplitudes (the partial pressure–volume maneuver), and large amplitudes (full‐range pressure–volume maneuvers) to quantify equivalent readouts, such as compliance, within different ranges of lung volumes.

**Results:**

The differences between saline‐ and HDM‐exposed mice increase with the maneuver amplitude. In the long protocol, for example, the decreased compliance caused by HDM was on average 3.2% (*p* = 0.39), 6.6% (*p* = 0.060), and 37.7% (*p* < 0.0001) when tested with maneuvers of small, intermediate, and large amplitudes, respectively. In the short protocol, these values were 3.1% (*p* = 0.35), 5.5% (*p* = 0.16), and 35.3% (*p* < 0.0001), respectively.

**Conclusion:**

Lung maneuvers of large amplitudes can detect physiological alterations with a greater sensitivity in mouse models of asthma. These results are particularly useful for scientists using mouse models to test countermeasures, such as drugs, in asthma.

## Graphical abstract

Lung maneuvers of small amplitudes, such as the ones performed in oscillometry, are the current gold standard to probe lung mechanics in animals, but they are insensitive to detect baseline physiological alterations in mouse models of asthma. Herein, lung maneuvers of different amplitudes were compared in two mouse models of asthma. It is demonstrated that large‐amplitude maneuvers are markedly more sensitive than maneuvers of smaller amplitudes to detect physiological lung alterations.

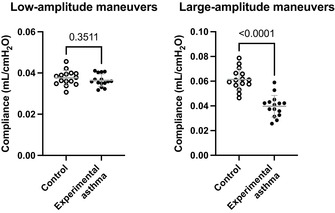

## 1 INTRODUCTION

Asthma prevalence has dwindled over the past three decades but remains high at approximately 3.3% globally.^1^ Projection studies have predicted no further decreases in the coming decades, indicating that asthma will remain an important medical, psychological, and financial burden.^1^ Mouse models will continue to be instrumental in advancing our understanding of asthma pathogenesis and in evaluating countermeasures (e.g., experimental drugs) to prevent or revert lung alterations that model those observed in asthma.^2^

Techniques and technologies for assessing physiological lung alterations in mice are also steadily evolving. Oscillometry is one such technique, providing almost instantaneous readouts that precisely assess many parameters of lung mechanics.^3^ It involves delivering small‐amplitude sinusoidal flow oscillation at different frequencies into the lungs and measuring the resulting pressure output to calculate impedance at each frequency together with its two components, resistance and reactance.^4^ Computational models can then be applied to the impedance spectrum to deduce readouts bearing more intuitive meanings, such as lung compliance and airway resistance.^4^ The advent of a commercial device, namely the flexiVent, has been a stepping stone, democratizing oscillometry by automating the whole process—from input signal delivery to output signal measurement, processing, and analyses.^3^

Unfortunately, oscillometric readouts are only minimally affected in mouse models of experimental asthma.^5^, ^6^, ^7^, ^8^, ^9^, ^10^, ^11^, ^12^, ^13^ This is inconvenient for many practical reasons, including the need to work with large numbers of mice. More sensitive readouts that swiftly measure physiological lung alterations in these models would hasten research and development.

New automated techniques using the flexiVent have recently been developed to probe the lungs over a wide range of volumes.^14^, ^15^ These maneuvers of larger amplitudes assess new readouts, such as total lung capacity and residual volume.^15^ However, they also allow similar readouts to be calculated, such as compliance.^15^ The readouts deduced from these new techniques were also shown to be more sensitive in detecting physiological lung alterations in a mouse model of acute lung injury induced by a single intranasal instillation of lipopolysaccharide.^16^ We hypothesized that they are also more sensitive in mouse models of asthma.

## 2 METHODS

### 2.1 Mice

A total of 90 female BALB/c mice (Charles River, Saint‐Constant, Canada) were studied. The protocols started when the mice were 8 weeks of age.

### 2.2 Experimental asthma

**FIGURE 1:**
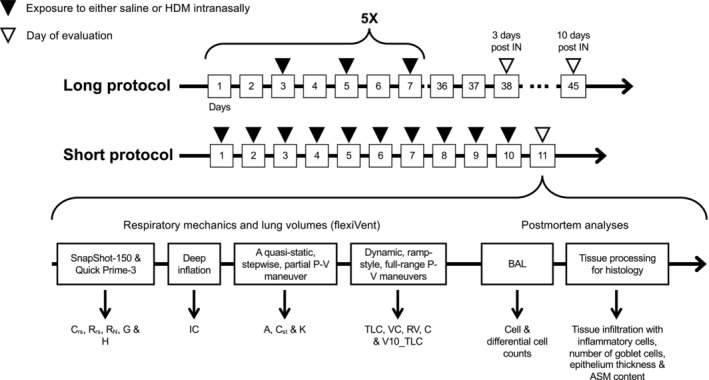
Schematic depicting the long and short protocols, as well as the sequence of interventions on testing days. See Section 2 for further details. A, the parameter A of Salazar‐Knowles equation (a proxy for the inspiratory capacity); ASM, airway smooth muscle; BAL, bronchoalveolar lavages; C, compliance; C_rs_, compliance of the respiratory system; C_st_, quasi‐static compliance; G, lung tissue resistance; H, lung tissue elastance; HDM, house dust mite; IN, intranasal instillation; K, the parameter K of Salazar‐Knowles equation (a volume‐independent indicator of the lung tissue compliance); P‐V, pressure–volume; R_N_, Newtonian resistance; R_rs_, resistance of the respiratory system; RV, residual volume; TLC, total lung capacity; VC, vital capacity; V10_TLC, lung volume at 10 cmH_2_O expressed in percentage of TLC.

Mice were exposed repeatedly to house dust mite (HDM) to induce experimental asthma. Each exposure was performed under isoflurane anesthesia and involved intranasal instillation of 2 mg/mL of HDM extract (*D*e*rmatophagoides pteronyssinus*, lot number 360923; Greer, Lenoir, NC, USA) in 25 μL of saline. The endotoxin concentration in the HDM extract was 47.3 EU per milligram. Control mice without experimental asthma were instilled with 25 μL of saline following the same exposure regimen. The acute and long protocols are illustrated in Figure 1. For the long protocol, exposures were performed thrice per week for 5 weeks. For the acute protocol, exposures were performed once daily for 10 consecutive days, as previously described.^12^, ^13^, ^17^ For the long protocol, mice were tested either 3 or 10 days after the last exposure. The goal of the two postexposure time points was to compare the same readouts during an active phase of inflammation (3 days postexposure) and after inflammation had substantially resorbed (10 days postexposure). For the acute protocol, mice were tested the day after the last exposure.

### 2.3 Respiratory mechanics

Respiratory mechanics were measured using the flexiVent (FX Module 2, SCIREQ, Montreal, Canada). The experimental setup and the mechanical ventilation were previously described.^18^ The maneuvers described below were all performed using the flexiVent in the order illustrated in Figure 1.

#### 2.3.1 Lung maneuvers of small amplitudes

Oscillometry was used for probing the lungs with maneuvers of small amplitudes. It was performed using two input signals, as recently described.^18^ One input signal is called the SnapShot‐150 and calculates resistance (R_rs_) and compliance (C_rs_) of the respiratory system. The other input signal is called the Quick Prime‐3 and calculates Newtonian resistance (R_N_), tissue resistance (G), and tissue elastance (H). The maximal volume amplitudes of these oscillometric signals are adjusted for mouse weight but correspond to approximately 265 and 125 μL for the SnapShot‐150 and the Quick Prime‐3, respectively, for a 20‐g mouse.

#### 2.3.2 Lung maneuvers of intermediate amplitudes

The quasi‐static, stepwise, pressure‐controlled, partial pressure–volume (P‐V) maneuver, hereinafter called the partial P‐V maneuver, was used as a maneuver of intermediate amplitudes. It was done as previously described.^15^, ^18^, ^19^ It allows the calculation of (1) the parameter A of Salazar‐Knowles equation, which is a proxy for the inspiratory capacity; (2) the parameter K of Salazar‐Knowles equation, which is a volume‐independent indicator of lung tissue compliance^19^; and (3) the quasi‐static compliance (C_st_). The maximal volume amplitude from the partial P‐V maneuver is influenced by the compliance of the respiratory system and the initial lung volume and is the one caused by a pressure excursion from 3 to 40 cmH_2_O.

#### 2.3.3 Lung maneuvers of large amplitudes

The dynamic, ramp‐style, full‐range P‐V maneuvers (hereinafter called the full‐range P‐V maneuvers) were used as maneuvers of large amplitudes. They were performed as previously described,^15^, ^18^ with a modification that had also been previously described.^16^ They calculate the total lung capacity (TLC), residual volume (RV), vital capacity (VC), and compliance (C) of the respiratory system, as well as the volume of the lungs at 10 cmH_2_O expressed in percentage of TLC (V_10__TLC). The maximal volume amplitude from the full‐range P‐V maneuver is influenced by the compliance of the respiratory system but is the one caused by a pressure excursion from a near‐zero lung volume to 40 cmH_2_O.

### 2.4 Bronchoalveolar lavages

Bronchoalveolar lavages (BAL) were performed, as previously described,^20^ to determine the total number of inflammatory cells, as well as the relative proportion of macrophages, lymphocytes, neutrophils, and eosinophils.

### 2.5 Histology

Histologic alterations observed in mice exposed to HDM in the short protocol were previously characterized at several occasions.^12^, ^17^, ^21^, ^22^ These quantifications were not repeated in the present study. Only lungs from mice subjected to the long protocol were studied by histology. We initially planned to assess eight mice per group to confirm the establishment of experimental asthma. Tissue processing and slide scanning were previously described.^18^

Staining with hematoxylin and eosin (H&E) was performed, as previously described,^18^ to evaluate the inflammatory cell infiltration within the lung tissue. For each mouse, 15 nonoverlapping photomicrographs from four noncontiguous lung sections were blindly scored from zero (no inflammation) to 5 (very severe inflammation). Periodic acid–Schiff (PAS) staining with Alcian blue was performed, as previously described, to determine the number of goblet cells.^20^ For each mouse, three noncontiguous lung sections were analyzed, representing 6 to 15 airways per mouse (average: 10.1 ± 2.0). The immunostaining with alpha‐smooth muscle actin was used to quantify the content of airway smooth muscle. For this immunostaining, the slides were (1) incubated for 30 min at 95°C in sodium citrate buffer, pH 6.0; (2) incubated for 20 min in 10% goat serum; (3) washed thrice for 15 min in phosphate‐buffered saline (PBS); (4) incubated for 90 min with the primary antibody (anti‐alpha smooth muscle actin, a rabbit monoclonal antibody, Abcam [Danaher], Washington, USA) at a concentration of 0.095 μg/mL; (5) washed thrice for 10 min in PBS; (6) incubated for 60 min with the secondary antibody (anti‐rabbit IgG HRP‐linked antibody, Cell Signaling Technology, Massachusetts, USA) at a 1:1000 dilution; (7) washed thrice for 10 min in PBS; (8) rinsed for 30 s with Triton 1% in PBS; (9) incubated for 10 min with DAB (DAB Substrate Kit, Abcam [Danaher], Washington, USA); and (10) counterstained for 1 min with Gill III hematoxylin. Three noncontiguous lung sections were analyzed, representing 6 to 18 airways per mouse (average: 11.1 ± 3.1). The airway smooth muscle content was calculated by measuring its area, which was then divided by the square of the basement membrane perimeter.^23^ The epithelium thickness was also analyzed on the same airways and was calculated for each airway by measuring its area, which was then divided by the basement membrane perimeter.

Because there was no enlargement of the airway smooth muscle after our initial analyses of eight mice per group, we decided to evaluate it in the remaining seven mice. This is because this feature was previously reported in prolonged models of asthma,^24^, ^25^ though not always.^26^ The final sample size for the content of airway smooth muscle is therefore 15, each representing 6 to 18 airways per mouse with an average of 10.9 ± 2.8.

### 2.6 Statistics

Individual data are presented, together with means ± standard deviations (SD). In the short protocol, *t*‐tests were used to assess the effect of experimental asthma (saline vs. HDM) on each readout. In the long protocol, two‐way analyses of variance (ANOVAs) were used to assess the effect of experimental asthma (saline vs. HDM), the time of testing post‐HDM exposure (3 vs. 10 days), and their interaction on each readout. When the interaction was significant, pairwise comparisons between groups were analyzed with a Tukey's multiple comparisons test. Relationships between the severity of experimental asthma, quantified by the total cells/mL of BAL or the percentage of eosinophils in BAL, with readouts from the large‐amplitude maneuvers, were investigated using simple linear regressions. All statistical analyses were performed using Prism (version 10.2.1, GraphPad, San Diego, CA, USA), except for power calculations, which were performed in R. Differences with a *p* < 0.05 were considered significant.

## 3 RESULTS

### 3.1 Inflammation

**FIGURE 2:**
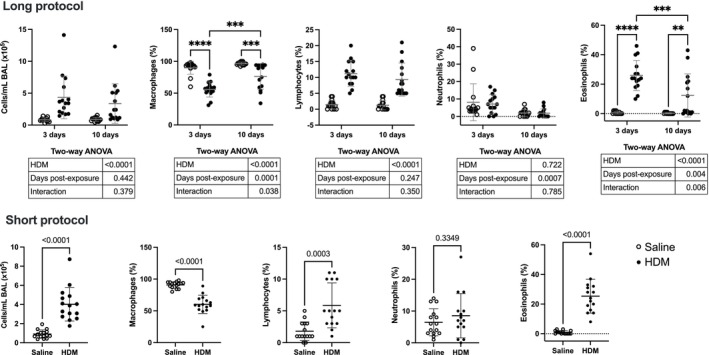
Inflammation in bronchoalveolar lavages (BAL). Cell counts were quantified in mice subjected to the long protocol 3 or 10 days after the last exposure to either saline (open circles) or house dust mite (HDM) (solid circles), or in mice subjected to the short protocol the day after the last exposure. Statistics for the long protocol are based on two‐way analyses of variance (ANOVAs). The results are provided underneath each graph. When the interaction was significant, a Tukey's multiple comparisons test was conducted. Asterisks indicate significant differences (**, ***, and **** signify *p* < 0.01, 0.001, and 0.0001, respectively). Statistics for the short protocol are based on *t*‐tests, and the *p*‐value is indicated above each comparison. *N* = 15 mice.

The inflammatory cells in BAL are depicted in Figure 2. HDM increased the total number of cells/mL of BAL in both the short and long protocols. In both protocols, HDM also increased the percentage of lymphocytes and eosinophils. This came at the expense of a decreased percentage of macrophages. The percentage of neutrophils was not affected by HDM in either protocol. Yet, a light neutrophilia was observed in both saline‐ and HDM‐exposed mice on day 3 in the long protocol and in the short protocol, which was likely due to the intranasal instillations (i.e., the sham procedure) and not due to the HDM exposure.^27^ This significantly receded by 10 days postexposure in the long protocol.

In the long protocol, there was no interaction between experimental asthma (i.e., saline vs. HDM exposure) and the postexposure interval (i.e., 3 vs. 10 days), indicating that the extra 7 days of recovery did not significantly decrease the total number of cells/mL of BAL. However, there were significant interactions for the percentage of eosinophils and macrophages, indicating that the nature of inflammation changed over the recovery period. More precisely, although the percentage of eosinophils decreased from 3 to 10 days of recovery, the percentage of macrophages increased.

### 3.2 Histology

**FIGURE 3:**
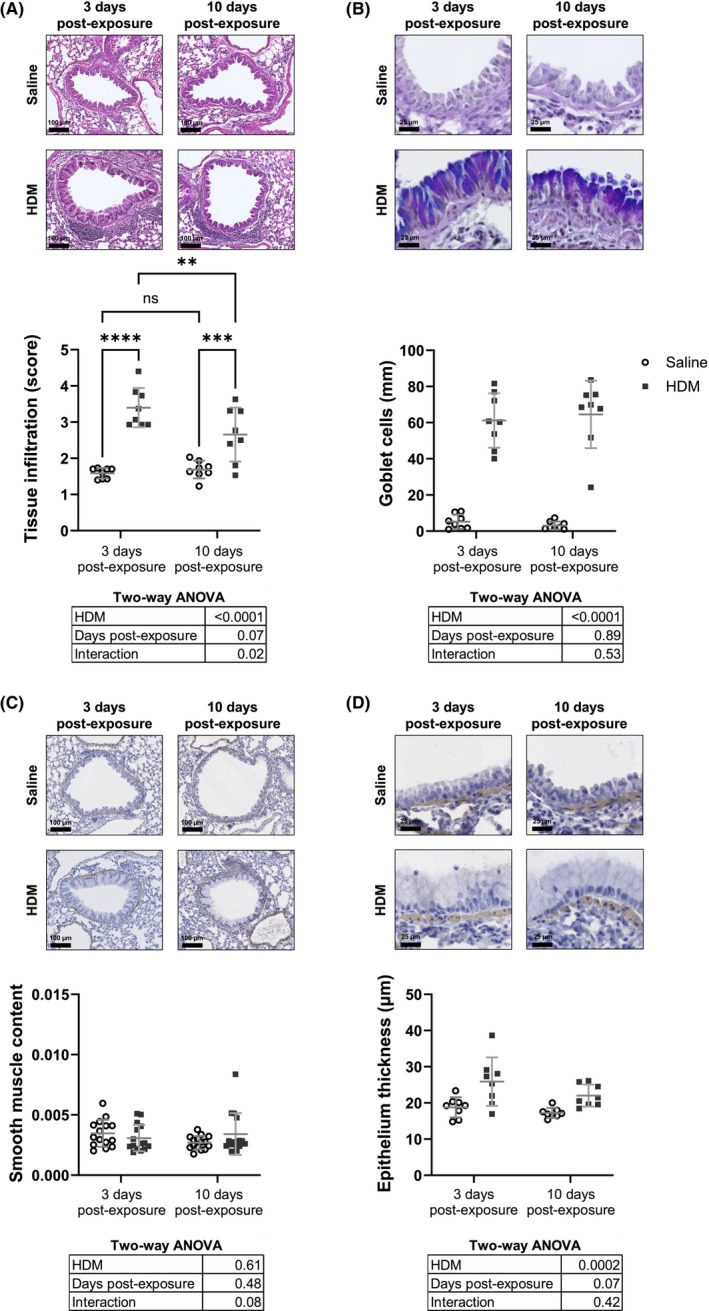
Histology. The tissue infiltration with inflammatory cells (A), the number of goblet cells. Scale bar: 100 µm (B), the content of airway smooth muscle. Scale bar: 25 µm (C), and the thickness of the airway epithelium. Scale bar: 100 µm (D) were quantified by histology in mice subjected to the long protocol 3 or 10 days after the last exposure to either saline (open circles) or house dust mite (HDM) (solid circles). Statistics are based on two‐way analyses of variance (ANOVAs). Scale bar: 25 µm The results are provided underneath each graph. When the interaction was significant, a Tukey's multiple comparisons test was conducted. Asterisks indicate significant differences (**, ***, and **** signify *p* < 0.01, *p* < 0.001, and *p* < 0.0001, respectively). *N* = 8 mice for A, B, and D, but 15 mice for C.

Histology on lung slices is depicted in Figure 3. Histology was performed only in mice subjected to the long protocol, as we had previously conducted these quantifications repeatedly for the short protocol.^12^, ^17^, ^21^, ^22^ HDM increased the tissue infiltration with inflammatory cells, the number of goblet cells, and the thickness of the epithelium, but did not affect the content of airway smooth muscle. There was also a significant interaction between experimental asthma and the postexposure interval for the tissue infiltration with inflammatory cells, indicating that the extra 7 days of recovery significantly decreased the level of cell infiltration within the lung tissue.

### 3.3 Physiological lung alterations in the long protocol

**FIGURE 4:**
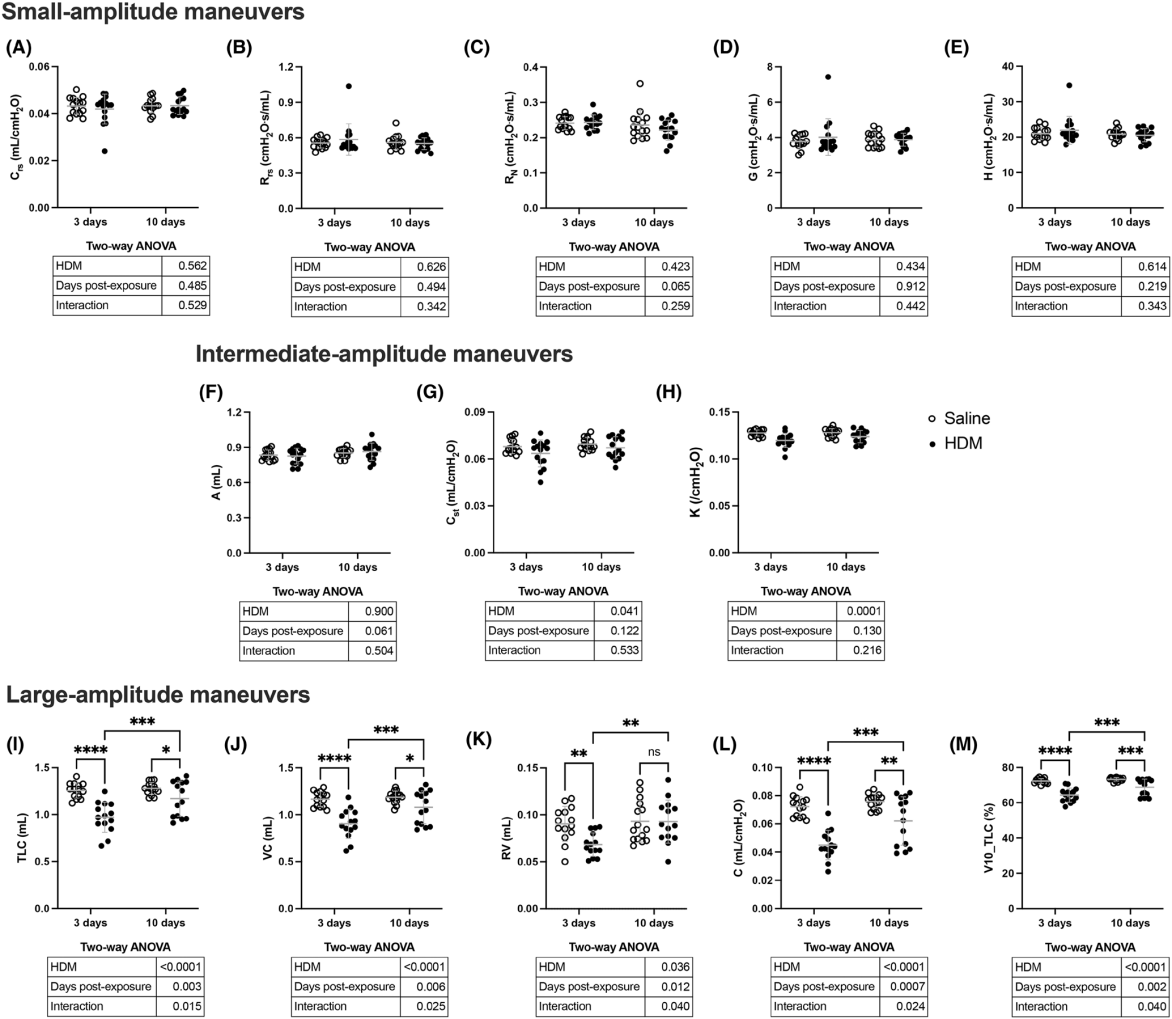
Physiological lung alterations in mice subjected to the long protocol. Readouts from the small‐amplitude maneuvers are shown in the upper part of the figure. (A–E) They are compliance of the respiratory system (C_rs_), resistance of the respiratory system (R_rs_), Newtonian resistance (R_N_), lung tissue resistance (G), and lung tissue elastance (H). Readouts from the intermediate‐amplitude maneuvers are shown in the middle part of the figure. (F–H) They are the parameter A of Salazar‐Knowles equation (A), quasi‐static elastance (C_st_), and the parameter K of Salazar‐Knowles' equation (K). Readouts from the large‐amplitude maneuvers are shown in the lower part of the figure. (I–M) They are total lung capacity (TLC), vital capacity (VC), residual volume (RV), compliance (C), and lung volume at 10 cmH_2_O expressed in percentage of TLC (V10_TLC). Individual results are shown (open and black circles for mice exposed to saline and house dust mite [HDM], respectively), together with the mean and SD (gray). Statistics are based on two‐way analyses of variance (ANOVAs). The results are provided underneath each graph. When the interaction was significant, a Tukey's multiple comparisons test was conducted. Asterisks indicate significant differences (*, **, ***, and **** signify *p* < 0.05, *p* < 0.01, *p* < 0.001, and *p* < 0.0001, respectively). ns stands for nonsignificant. *N* = 15 mice.

The results in mice subjected to the long protocol are depicted in Figure 4. By probing the lungs with oscillometry, which imposes small‐amplitude maneuvers, no physiological alterations were detected in mice exposed to HDM, irrespective of whether they were studied 3 or 10 days postexposure. This was true for each oscillometric readout (C_rs_, R_rs_, R_N_, G, and H).

By probing the lungs with the partial P‐V maneuver, which is an intermediate‐amplitude maneuver, no significant differences in A were detected. This indicates that the total amount of air entering the lungs from an inflating pressure of 3 to 40 cmH_2_O was not different between groups. However, HDM significantly decreased C_st_ and K, indicating that the lungs of HDM‐exposed mice were stiffer than the lungs of saline‐exposed mice. There were no interactions between experimental asthma and the postexposure interval for both C_st_ and K, indicating that the extra 7 days of recovery did not significantly affect these alterations.

By probing the lungs with the full‐range P‐V maneuvers, which are large‐amplitude maneuvers, every measured readout was significantly affected by HDM. More precisely, HDM decreased TLC, VC, RV, C, and V10_TLC. Decreases in TLC and VC essentially indicate that the total amount of air entering the degassed lungs from an inflating pressure of 0 to 40 cmH_2_O was reduced. A decreased RV indicates that a smaller amount of air remained trapped in the lungs following a deflation from a pressure of 40 to −10 cmH_2_O. And decreases in C and V10_TLC indicate stiffer lungs. These changes, expressed in percentage, are also shown in Table 1. All readouts derived from the large‐amplitude maneuvers were also linearly related to the severity of experimental asthma, assessed either by the total cells/mL of BAL or by the percentage of eosinophils in BAL. These results are shown in Table 2.

**TABLE 1:** Physiological changes induced by house dust mite, expressed in percentage.

Protocol	Long		Short
Postexposure interval	3 days	10 days	1 day
Readouts from small‐amplitude maneuvers			
C_rs_	−3.2	0.1	−3.1
R_rs_	5.4	−1.7	1.4
R_N_	1.0	−6.1	3.1
G	6.6	0.1	−0.5
H	4.4	−1.4	3.1
Readouts from intermediate‐amplitude maneuvers			
A	−1.0	1.4	−3.8
C_st_	−6.6	−3.5	−5.6
K	−6.2	−3.4	−1.7
Readouts from large‐amplitude maneuvers			
TLC	−22.7	−8.4	−26.7
VC	−22.5	−9.1	−26.6
RV	−24.4	−0.3	−27.7
C	−37.7	−18.3	−35.3
V10_TLC	−11.1	−6.2	−6.0

*Note*: All values are presented in percentage, relative to their respective control group subjected to the same exposure regimen but treated with saline instead of house dust mite.

Abbreviations: A, the parameter A of Salazar‐Knowles equation (a proxy for the inspiratory capacity); C, compliance; C_rs_, compliance of the respiratory system; C_st_, quasi‐static compliance; G, lung tissue resistance; H, lung tissue elastance; K, the parameter K of Salazar‐Knowles equation (a volume‐independent indicator of the lung tissue compliance); R_N_, Newtonian resistance; R_rs_, resistance of the respiratory system; RV, residual volume; TLC, total lung capacity; VC, vital capacity; V10_TLC, lung volume at 10 cmH_2_O expressed in percentage of TLC.

**TABLE 2:** Relationships between the severity of experimental asthma and readouts from the large‐amplitude maneuvers.

Experimental asthma severity	Physiological readouts	Long protocol		Short protocol	
		*R* ^2^	*p*‐value	*R* ^2^	*p*‐value
Total cells/mL of BAL versus	TLC	0.50	<0.0001	0.28	0.003
	VC	0.50	<0.0001	0.26	0.004
	RV	0.12	0.009	0.29	0.002
	C	0.50	<0.0001	0.67	0.0004
	V10_TLC	0.40	<0.0001	0.57	<0.0001
% of eosinophils in BAL versus	TLC	0.65	<0.0001	0.45	<0.0001
	VC	0.63	<0.0001	0.43	<0.0001
	RV	0.22	0.0003	0.40	0.0002
	C	0.72	<0.0001	0.48	<0.0001
	V10_TLC	0.69	<0.0001	0.45	<0.0001

Abbreviations: BAL, bronchoalveolar lavages; C, compliance; RV, residual volume; TLC, total lung capacity; VC, vital capacity; V10_TLC, lung volume at 10 cmH_2_O expressed in percentage of TLC.

The postexposure interval also significantly affected all readouts of the full‐range P‐V maneuvers. There were also significant interactions between experimental asthma and the postexposure interval, which were again observed across all measured readouts. This indicates that differences between mice studied at 3 and 10 days postexposure depended on whether they were exposed to saline or HDM. Post‐hoc analyses demonstrated that TLC, VC, RV, C, and V10_TLC were all significantly lower at 3 versus 10 days postexposure in HDM‐exposed mice, indicating that lung alterations induced by HDM were recovering.

### 3.4 Physiological lung alterations in the short protocol

**FIGURE 5:**
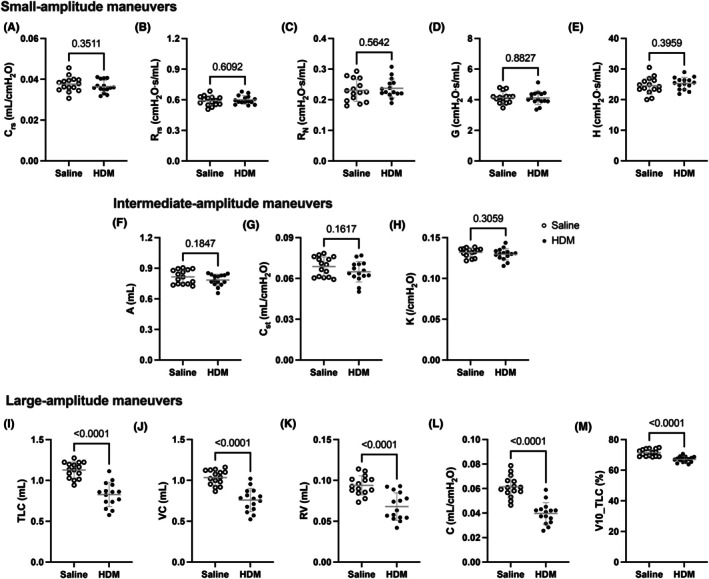
Physiological lung alterations in mice subjected to the short protocol. Readouts from the small‐amplitude maneuvers are shown in the upper part of the figure. (A–E) They are compliance of the respiratory system (C_rs_), resistance of the respiratory system (R_rs_), Newtonian resistance (R_N_), lung tissue resistance (G), and lung tissue elastance (H). Readouts from the intermediate‐amplitude maneuvers are shown in the middle part of the figure. (F–H) They are the parameter A of Salazar‐Knowles equation (A), quasi‐static elastance (C_st_), and the parameter K of Salazar‐Knowles equation (K). Readouts from the large‐amplitude maneuvers are shown in the lower part of the figure. (I–M) They are total lung capacity (TLC), vital capacity (VC), residual volume (RV), compliance (C), and lung volume at 10 cmH_2_O expressed in percentage of TLC (V10_TLC). Individual results are shown (open and black circles for mice exposed to saline and house dust mite [HDM], respectively), together with the mean and standard deviation (SD) (gray). Statistics are based on *t*‐tests. The *p*‐value is indicated above each comparison. *N* = 15 mice.

The results of mice subjected to the short protocol are depicted in Figure 5. The results were similar to the effect of HDM in the long protocol. By probing the lungs either with small or intermediate‐amplitude maneuvers, there were no differences between groups. In contrast, when the lungs were probed with maneuvers of large amplitudes, every measured readout was significantly reduced by HDM, indicating again stiffer lungs with less volume available for entering air. These changes, expressed in percentage, are also shown in Table 1. As per the long protocol, all readouts derived from the large‐amplitude maneuvers in the short protocol were also linearly related to the severity of experimental asthma, assessed either by the total cells/mL of BAL or by the percentage of eosinophils in BAL. These results are shown in Table 2.

## 4 DISCUSSION

This study was designed to compare the sensitivity of lung maneuvers of different amplitudes in detecting physiological alterations in mouse models of asthma. These comparisons were made in female BALB/c mice using a sho and a prolonged model of asthma induced by repeated intranasal exposures to HDM. The results indicated that lung maneuvers of large amplitudes are markedly more sensitive than small and intermediate amplitudes in detecting physiological alterations in mouse models of asthma.

In human asthma, readouts obtained by oscillometry are clearly abnormal,^28^, ^29^, ^30^, ^31^, ^32^, ^33^, ^34^, ^35^, ^36^, ^37^, ^38^ and they are usually associated with the severity of asthma.^29^, ^34^, ^39^ As alluded to in the Introduction, this is not the case in mouse models of asthma.^5^, ^6^, ^7^, ^8^, ^9^, ^10^, ^11^, ^12^, ^13^ This interspecies difference may stem from different reasons, but the most likely reason is the absence versus the presence of large lung maneuvers prior to oscillometric measurements. Indeed, according to current human guidelines,^40^ deep inspirations and forceful expiratory maneuvers, such as the ones required in spirometry, should be avoided prior to oscillometry. This is because these maneuvers typically tend to renormalize lung mechanics and would thus interfere with the detection of alterations in asthmatic patients. In contrast, optimal mechanical ventilation protocols in mice include frequent deep inflations, also called recruitment maneuvers, which have been shown to improve lung mechanics and minimize lung injury during mechanical ventilation.^41^ These recruitment maneuvers were also recommended because they reduce variability within and between subjects.^42^ Over time, imposing recruitment maneuvers prior to the measurement of lung mechanics by oscillometry in mice became common practices. It is hard to pinpoint exactly why. It is probably because the initial goal was to assess the mechanics of the lung tissue, and the recruitment maneuvers were then limiting the confounding effects of airway closure and air trapping on lung mechanics.^42^ Yet, because an important goal in animal research is to deepen our understanding of human diseases, it is strange that current recommendations regarding the usage of large maneuvers prior to oscillometry diverge diametrically between mice and humans. Perhaps these common practices in mice should be revisited. It is clear based on other techniques, such as the double‐chamber plethysmography, that mice with experimental asthma exhibit physiological lung alterations.^13^ In the present study, oscillometry was done conventionally after recruitment maneuvers, but future studies will be needed to assess the impact of recruitment maneuvers on physiological lung alterations in mice with experimental asthma.

The present study confirmed that oscillometry, at least when performed after recruitment maneuvers, is unable to detect alterations in mice with experimental asthma.^13^ In contradistinction, the partial P‐V maneuver, which is a maneuver of intermediate amplitude, was successful to detect decreases in C_st_ and K, at least in the long protocol of HDM exposure. Both of these readouts were also numerically decreased in the short protocol, but not significantly. It is worth mentioning that this lack of significant effect may be due to a reduced statistical power. Indeed, because there was only one day of evaluation postexposure in the short protocol (1 day postexposure), instead of two in the long protocol (3 and 10 days postexposure), the total number of mice per conditions (saline vs. HDM) were half the ones in the long protocol. In fact, when mice evaluated on days 3 and 10 after the last exposure in the long protocol were analyzed separately using *t*‐tests, C_st_ was no longer different between saline and HDM‐exposed mice. K was still different though, both at 3 and 10 days after the last exposure (*p* = 0.0008 and 0.044, respectively). Regardless, the significant differences observed in the long protocol still suggested that a lung maneuver of intermediate amplitude is more sensitive than oscillometry in detecting physiological alterations.

More impressively, the full‐range P‐V maneuvers, which are maneuvers of large amplitudes, discriminated very well between groups of mice with and without experimental asthma. The differences were highly significant for all readouts in both the long and short protocols. The results of each readout in the long protocol also demonstrated a significant interaction between experimental asthma (saline vs. HDM) and the postexposure interval (3 vs. 10 days). This suggests that these readouts have the sensitivity to discern groups of HDM‐exposed mice that only differ by the number of days that they have been last exposed (3 vs. 10 days). This is remarkable considering that the number of inflammatory cells in BAL did not change significantly over this period (Figure 2); although qualitative changes in the nature of BAL inflammation were detected (a greater percentage of macrophages with a lower percentage of eosinophils 10 vs. 3 days after the last exposure), and the level of tissue infiltration with inflammatory cells also decreased significantly (Figure 3). Sample size analyses also suggested that, for all readouts from the large‐amplitude maneuvers, three to nine mice would be sufficient to obtain 80% power for detecting a significant difference (*p* < 0.05) between mice with and without experimental asthma (Table 3). Even when studying a countermeasure (e.g., an experimental drug) that would inhibit these alterations by only about 30%, it would mean that it can be done with a practicable number of mice.

**TABLE 3:** Sample sizes to obtain significant differences (*p* < 0.05) with 80% power between saline‐ and HDM‐exposed mice.

Protocol	Long		Short
Postexposure interval	3 days	10 days	1 day
Readouts from small‐amplitude maneuvers			
C_rs_	208	–	132
R_rs_	135	–	–
R_N_	–	–	–
G	127	–	–
H	148	–	156
Readouts from intermediate‐amplitude maneuvers			
A	–	–	64
C_st_	36	86	58
K	9	27	116
Readouts from large‐amplitude maneuvers			
TLC	5	23	4
VC	5	21	5
RV	9	–	6
C	3	12	4
V10_TLC	4	8	5

*Note*: A hyphen signifies that the calculated sample was beyond 250.

Abbreviations: A, the parameter A of Salazar‐Knowles equation (a proxy for the inspiratory capacity); C, compliance; C_rs_, compliance of the respiratory system; C_st_, quasi‐static compliance; G, lung tissue resistance; H, lung tissue elastance; HDM, house dust mite; K, the parameter K of Salazar‐Knowles equation (a volume‐independent indicator of the lung tissue compliance); R_N_, Newtonian resistance; R_rs_, resistance of the respiratory system; RV, residual volume; TLC, total lung capacity; VC, vital capacity; V10_TLC, lung volume at 10 cmH_2_O expressed in percentage of TLC.

Why the amplitude of lung maneuvers matters? The answer to this question is uncertain. Perhaps alterations caused by experimental asthma are only perceptible at high volumes. Therefore, probing the lungs by small‐amplitude maneuvers (i.e., oscillometry) at a volume near functional residual capacity may not stretch the lung sufficiently to detect these alterations.

But then why the large‐amplitude maneuvers are more sensitive than the intermediate‐amplitude maneuvers, as both probe the lungs all the way to 40 cmH_2_O? Again, the answer to this question is speculative. Perhaps the initial level of lung inflation is important. For the partial P‐V maneuver, the lungs are initially set to a positive pressure of 3 cmH_2_O (determined by the positive end‐expiratory pressure). Yet, this does not mean that the initial level of lung inflation was the same in every mouse. Stiffer lungs, for example, were presumably less inflated at 3 cmH_2_O than more compliant lungs. The amount of air entering into the stiffer lungs between 3 and 40 cmH_2_O may then have been similar, even if their actual maximal inflation was lower, simply because their initial level of inflation was also lower. This may have been the case in mice exposed to HDM, explaining the lack of difference in the parameter A. It would also explain the smaller‐than‐expected differences in C_st_ and K, as these would then have been measured over a lower range of lung inflation.

In contrast, the initial level of lung inflation during the full‐range P‐V maneuvers is very well controlled. The volume of air in the lungs at the beginning of the first inflating maneuver is always theoretically zero, as it starts after a period of degassing (see Section 2). For stiffer lungs in this context, not only the amount of air entering from 0 to 40 cmH_2_O will be less (decreasing TLC and VC), but the change in volume per unit of pressure will also be less (decreasing C and V10_TLC). Because these are essentially the results obtained in mice with experimental asthma for both the short and long protocols, it is tempting to conclude that mice with experimental asthma have stiffer lungs.

Finally, why would lungs be stiffer in mouse models of asthma, and why to a similar extent between the short and long protocols? Because the extent of the physiological lung alterations was about the same in the short and long protocols, and some of these alterations receded with an additional 7 days of recovery in mice subjected to the long protocol, the more likely explanation is consolidation. Tissue edema, combined with the accumulation of inflammatory, exudative, and mucosal fluid within the airspaces (airways and alveoli), presumably forces the lungs to work at a higher physical volume. Indeed, the lung tissue is likely to be stretched not only by the amount of air within the lungs but also by the fluid accumulating in them. The volume loss by the accumulation of fluid would obviously steal the first and the steepest part of the P‐V curve, which is inherently more compliant. The part remaining for air (which is the part measured by the flexiVent during the full‐range P‐V maneuvers) would then appear smaller (decreasing TLC and VC) and stiffer (increasing C and V10_TLC). The excess of fluid stealing room that is typically available for air would also explain the decrease in RV.

## 5 CONCLUSION

This study demonstrates that lung maneuvers of large amplitudes can detect physiological alterations with a substantially greater sensitivity than maneuvers of small and intermediate amplitudes in mouse models of asthma. Although the mechanistic underpinnings are arguable, alterations that are only perceptible at higher lung volumes and/or a better control over the degree of lung inflation before the beginning of the large‐amplitude maneuvers may play a role. With such a sensitivity, readouts from the full‐range P‐V maneuvers are expected to be useful for many pragmatic reasons, including for testing countermeasures in preclinical models of asthma using sample sizes that align with the 3Rs principles in research.

## AUTHOR CONTRIBUTIONS

**Magali Boucher:** Conceptualization; data curation; formal analysis; investigation; methodology; project administration; writing – review and editing. **Cyndi Henry:** Conceptualization; data curation; formal analysis; funding acquisition; investigation; methodology; project administration; validation; visualization; writing – review and editing. **Marie‐Josée Beaulieu:** Data curation; formal analysis; writing – review and editing. **Andrés Rojas‐Ruiz:** Data curation; writing – review and editing. **Ynuk Bossé:** Conceptualization; funding acquisition; investigation; methodology; project administration; resources; software; supervision; validation; visualization; writing – original draft; writing – review and editing.

## FUNDING INFORMATION

This study was supported by the Natural Sciences and Engineering Research Council of Canada (NSERC, RGPIN‐2020‐06355, and ALLRP‐570485‐2021), the Canadian Institutes of Health Research (CIHR, 508356‐202209PJT), and the Fondation de l'IUCPQ (Institut Universitaire de Cardiologie et de Pneumologie de Québec).

## CONFLICT OF INTEREST STATEMENT

The authors have no conflicts of interest.

## ETHICS STATEMENT

All procedures were approved by the Committee of Animal Care of Université Laval following the guidelines from the Canadian Council on Animal Care (protocol 2024‐1602).

## Data Availability

The datasets used and analyzed during the current study are available from the corresponding author on reasonable request.
